# Supplementary material for: Familial and Parental Predictors of Physical Activity in Late Adolescence: Prospective Analysis over a Two-Year Period
Source: Healthcare (Basel). 2021 Jan 29;9(2):132. doi: 10.3390/healthcare9020132 (PMC7912008; doi:10.3390/healthcare9020132)
Supplement: Supplementary file 1 [file healthcare-09-00132-s001.pdf]

Table S1

Differences between participants based on sufficiency/insufficiency of physical activity levels (PAL) at baseline

| | Baseline | | | | Mann Whitney/ $\chi^2$ (p) |
|---|---|---|---|---|---|
| | Insufficient PAL | | Sufficient PAL | | |
| | F | % | F | % | |
| Gender $\chi^2$ | | | | | 103.1 (0.001) |
| Males | 192 | 39.6 | 220 | 78.3 | |
| Females | 286 | 59.0 | 61 | 21.7 | |
| Missing | 7 | 1.4 | 0 | 0.0 | |
| | | | | | |
| Socioeconomic status | | | | | 0.14 (0.88) |
| Below average | 5 | 1.0 | 8 | 2.8 | |
| Average | 456 | 94.0 | 255 | 90.7 | |
| Above average | 24 | 4.9 | 18 | 6.4 | |
| Missing | 0 | 0.0 | 0 | 0.0 | |
| | | | | | |
| Paternal education | | | | | 4.18 (0.001) |
| Elementary school | 40 | 8.2 | 13 | 4.6 | |
| High school | 369 | 76.1 | 190 | 67.6 | |
| College level | 42 | 8.7 | 43 | 15.3 | |
| University level | 34 | 7.0 | 35 | 12.5 | |
| Missing | 0 | 0.0 | 0 | 0.0 | |
| | | | | | |
| Maternal education | | | | | 2.15 (0.03) |
| Elementary school | 149 | 30.7 | 65 | 23.1 | |
| High school | 279 | 57.5 | 180 | 64.1 | |
| College level | 26 | 5.4 | 18 | 6.4 | |
| University level | 29 | 6.0 | 18 | 6.4 | |
| Missing | 2 | 0.4 | 0 | 0.0 | |
| | | | | | |
| Parental/familial conflict | | | | | 5.21 (0.001) |
| Almost never | 161 | 33.2 | 142 | 50.5 | |
| Rarely | 198 | 40.8 | 98 | 34.9 | |
| From time to time | 104 | 21.4 | 41 | 14.6 | |
| Often | 22 | 4.5 | | | |
| Missing | 0 | 0.0 | 0 | 0.0 | |
| | | | | | |
| Parental absence | | | | | 0.68 (0.49) |
| Always at home | 109 | 22.5 | 41 | 14.6 | |
| Rarely absent | 104 | 21.4 | 82 | 29.2 | |
| Occasionally absent | 168 | 34.6 | 105 | 37.4 | |

| | | | | | |
|---|---|---|---|---|---|
| Often absent | 102 | 21.0 | 53 | 18.9 | |
| Missing | 2 | 0.4 | 0 | 0.0 | |
| | | | | | |
| Parental care | | | | | 2.39 (0.02) |
| Parents do not care at all | 0 | 0.0 | 8 | 2.8 | |
| Do not care | 12 | 2.5 | 8 | 2.8 | |
| Good care | 159 | 32.8 | 105 | 37.4 | |
| Very much care | 314 | 64.7 | 160 | 56.9 | |
| Missing | 0 | 0.0 | 0 | 0.0 | |
| | | | | | |
| Parental questioning | | | | | 0.08 (0.92) |
| Mostly never | 20 | 4.1 | 8 | 2.8 | |
| Rarely | 60 | 12.4 | 38 | 13.5 | |
| From time to time | 174 | 35.9 | 103 | 36.7 | |
| Often/regularly | 229 | 47.2 | 132 | 47.0 | |
| Missing | 2 | 0.4 | 0 | 0.0 | |
| | | | | | |
| Sport participation $^{x2}$ | | | | | 98.02 (0.001) |
| Yes, currently | 43 | 8.9 | 98 | 34.9 | |
| Yes, but quit | 213 | 43.9 | 125 | 44.5 | |
| No, never | 227 | 46.8 | 58 | 20.6 | |
| Missing | 2 | 0.4 | 0 | 0.0 | |

$^{x2}$ indicates variables where differences between groups were calculated by $\chi^2$ test

Table S2

Differences between participants based on sufficiency/insufficiency of physical activity levels (PAL) at first follow-up measurement

| | Baseline | | | | Mann Whitney/ $\chi^2$ (p) |
|---|---|---|---|---|---|
| | Insufficient PAL | | Sufficient PAL | | |
| | F | % | F | % | |
| Gender $\chi^2$ | | | | | 57.45 (0.01) |
| Males | 284 | 46.7 | 128 | 81.0 | |
| Females | 317 | 52.1 | 30 | 19.0 | |
| Missing | 7 | 1.2 | 0 | 0.0 | |
| | | | | | |
| Socioeconomic status | | | | | 0.33 (0.73) |
| Below average | 11 | 1.8 | 2 | 1.3 | |
| Average | 564 | 92.8 | 147 | 93.0 | |
| Above average | 33 | 5.4 | 9 | 5.7 | |
| Missing | 0 | 0.0 | 0 | 0.0 | |
| | | | | | |
| Paternal education | | | | | 1.20 (0.22) |
| Elementary school | 42 | 6.9 | 11 | 7.0 | |
| High school | 449 | 73.8 | 110 | 69.6 | |
| College level | 71 | 11.7 | 14 | 8.9 | |
| University level | 46 | 7.6 | 23 | 14.6 | |
| Missing | 0 | 0.0 | 0 | 0.0 | |
| | | | | | |
| Maternal education | | | | | 1.63 (0.10) |
| Elementary school | 180 | 29.6 | 34 | 21.5 | |
| High school | 354 | 58.2 | 105 | 66.5 | |
| College level | 32 | 5.3 | 12 | 7.6 | |
| University level | 40 | 6.6 | 7 | 4.4 | |
| Missing | 2 | 0.3 | 0 | 0.0 | |
| | | | | | |
| Parental/familial conflict | | | | | 2.25 (0.02) |
| Almost never | 230 | 37.8 | 73 | 46.2 | |
| Rarely | 237 | 39.0 | 59 | 37.3 | |
| From time to time | 121 | 19.9 | 24 | 15.2 | |
| Often | 20 | 3.3 | 2 | 1.3 | |
| Missing | 0 | 0.0 | 0 | 0.0 | |
| | | | | | |
| Parental absence | | | | | 0.49 (0.61) |
| Always at home | 126 | 20.7 | 24 | 15.2 | |
| Rarely absent | 143 | 23.5 | 43 | 27.2 | |
| Occasionally absent | 206 | 33.9 | 67 | 42.4 | |

| | | | | | |
|---|---|---|---|---|---|
| Often absent | 133 | 21.9 | 22 | 13.9 | |
| Missing | 0 | 0.0 | 2 | 1.3 | |
| | | | | | |
| Parental care | | | | | 1.21 (0.22) |
| Parents do not care at all | 4 | 0.7 | 4 | 2.5 | |
| Do not care | 18 | 3.0 | 2 | 1.3 | |
| Good care | 203 | 33.4 | 61 | 38.6 | |
| Very much care | 383 | 63.0 | 91 | 57.6 | |
| Missing | 0 | 0.0 | 0 | 0.0 | |
| | | | | | |
| Parental questioning | | | | | 1.02 (0.30) |
| Mostly never | 24 | 3.9 | 4 | 2.5 | |
| Rarely | 70 | 11.5 | 28 | 17.7 | |
| From time to time | 221 | 36.3 | 56 | 35.4 | |
| Often/regularly | 291 | 47.9 | 70 | 44.3 | |
| Missing | 2 | 0.3 | 0 | 0.0 | |
| | | | | | |
| Sport participation $^{\chi 2}$ | | | | | 103.94 (0.001) |
| Yes, currently | 70 | 11.5 | 71 | 44.9 | |
| Yes, but quit | 274 | 45.1 | 64 | 40.5 | |
| No, never | 262 | 43.1 | 23 | 14.6 | |
| Missing | 2 | 0.3 | 0 | 0.0 | |

$^{\chi 2}$ indicates variables where differences between groups were calculated by $\chi^2$ test

Table S3

Differences between participants based on sufficiency/insufficiency of physical activity levels (PAL) at second follow-up measurement

| | Baseline | | | | Mann Whitney/ $\chi^2$ (p) |
|---|---|---|---|---|---|
| | Insufficient PAL | | Sufficient PAL | | |
| | F | % | F | % | |
| Gender $\chi^2$ | | | | | 74.74 (0.001) |
| Males | 260 | 45.2 | 152 | 79.6 | |
| Females | 313 | 54.4 | 34 | 17.8 | |
| Missing | 2 | 0.3 | 5 | 2.6 | |
| | | | | | |
| Socioeconomic status | | | | | 0.67 (0.49) |
| Below average | 7 | 1.2 | 6 | 3.1 | |
| Average | 537 | 93.4 | 174 | 91.1 | |
| Above average | 31 | 5.4 | 11 | 5.8 | |
| Missing | 0 | 0.0 | 0 | 0.0 | |
| | | | | | |
| Paternal education | | | | | 1.98 (0.09) |
| Elementary school | 43 | 7.5 | 10 | 5.2 | |
| High school | 425 | 73.9 | 134 | 70.2 | |
| College level | 61 | 10.6 | 24 | 12.6 | |
| University level | 46 | 8.0 | 23 | 12.0 | |
| Missing | 0 | 0.0 | 0 | 0.0 | |
| | | | | | |
| Maternal education | | | | | 0.30 (0.70) |
| Elementary school | 165 | 28.7 | 49 | 25.7 | |
| High school | 337 | 58.6 | 122 | 63.9 | |
| College level | 29 | 5.0 | 15 | 7.9 | |
| University level | 42 | 7.3 | 5 | 2.6 | |
| Missing | 2 | 0.3 | 0 | 0.0 | |
| | | | | | |
| Parental/familial conflict | | | | | 4.34 (0.001) |
| Almost never | 204 | 35.5 | 99 | 51.8 | |
| Rarely | 230 | 40.0 | 66 | 34.6 | |
| From time to time | 121 | 21.0 | 24 | 12.6 | |
| Often | 20 | 3.5 | 2 | 1.0 | |
| Missing | 0 | 0.0 | 0 | 0.0 | |
| | | | | | |
| Parental absence | | | | | 1.08 (0.27) |
| Always at home | 119 | 20.7 | 31 | 16.2 | |
| Rarely absent | 143 | 24.9 | 43 | 22.5 | |
| Occasionally absent | 191 | 33.2 | 82 | 42.9 | |

| | | | | | |
|---|---|---|---|---|---|
| Often absent | 120 | 20.9 | 35 | 18.3 | |
| Missing | 2 | 0.3 | 0 | 0.0 | |
| | | | | | |
| Parental care | | | | | 2.09 (0.04) |
| Parents do not care at all | 6 | 1.0 | 2 | 1.0 | |
| Do not care | 16 | 2.8 | 4 | 2.1 | |
| Good care | 184 | 32.0 | 80 | 41.9 | |
| Very much care | 369 | 64.2 | 105 | 55.0 | |
| Missing | 0 | 0.0 | 0 | 0.0 | |
| | | | | | |
| Parental questioning | | | | | 0.09 (0.92) |
| Mostly never | 24 | 4.2 | 4 | 2.1 | |
| Rarely | 71 | 12.3 | 27 | 14.1 | |
| From time to time | 205 | 35.7 | 72 | 37.7 | |
| Often/regularly | 273 | 47.5 | 88 | 46.1 | |
| Missing | 2 | 0.3 | 0 | 0.0 | |
| | | | | | |
| Sport participation $^{x2}$ | | | | | 89.85 (0.001) |
| Yes, currently | 65 | 11.3 | 76 | 39.8 | |
| Yes, but quit | 259 | 45.0 | 79 | 41.4 | |
| No, never | 251 | 43.7 | 34 | 17.8 | |
| Missing | 0 | 0.0 | 2 | 1.0 | |

$^{x2}$ indicates variables where differences between groups were calculated by $\chi^2$ test
